# Reduction of operator radiation exposure using a passive robotic device during fluoroscopy-guided arterial puncture: an experimental study in a swine model

**DOI:** 10.1186/s41747-019-0098-1

**Published:** 2019-05-29

**Authors:** Muhammad Umair Ahmad Khan, Chang-Hwan Yoon, Byung-Ju Yi

**Affiliations:** 1 Department of Mechatronics Engineering, Hanyang University, 55 Hanyangdeahak-ro, Sangnok-gu, Ansan, 15588 Gyeonggi-do South Korea; 2 Department of Internal Medicine, Seoul National University Bundang Hospital, 82, Gumi-ro 173 Beon-gil, Bundang-gu, Seongnam, 13620 Gyeonggi-do South Korea; 3 Department of Electronic Systems Engineering, Hanyang University, 55 Hanyangdeahak-ro, Sangnok-gu, Ansan, 15588 Gyeonggi-do South Korea

**Keywords:** Fluoroscopy, Occupational exposure, Radiation exposure, Radiation protection, Radiology (interventional)

## Abstract

**Background:**

Vascular interventions imply radiation exposure to the operating physician (OP). To reduce radiation exposure, we propose a novel passive robotic device for fluoroscopy-guided arterial puncturing.

**Methods:**

X-ray dose rates were measured for a total of 30 fluoroscopy-guided puncture femoral arteries in 15 pigs. Fifteen punctures were performed with the device while the other 15 were performed without the device by an interventional cardiologist with 10 years of experience. Parametric *t* test was used.

**Results:**

The success rate with the device was 100%. Overall, the OP received more radiation (0.41 mSv/h) as compared to the assistant (0.06 mSv/h) (*p* <  0.001) and, amongst OP’s body parts, hands received more radiation than other body parts (*p* <  0.001). The radiation dose rate to the OP’s hands during arterial puncturing performed manually without the device was 0.95 ± 0.25 mSv/h whereas it was 0.14 ± 0.006 mSv/h using the device, resulting in an 85% reduction (*p* <  0.001). For the head, the dose was reduced from 0.16 mSv/h to 0.08 mSv/h (50% reduction, *p* <  0.001), and for the dominant arm, from 0.12 mSv/h to 0.07 mSv/h (42% reduction, *p* <  0.001). The fluoroscopy time was reduced from 4.5 ± 0.15 min to 4.3 ± 0.11 min device (*p* = 0.002).

**Conclusions:**

In a swine model, fluoroscopy time and radiation exposure for the OP puncturing femoral artery were significantly reduced by using the passive robotic device.

## Key points

- A passive robotic device for arterial puncturing was developed.
- Using the robotic device, the mean fluoroscopy time was significantly reduced from 4.5 to 4.3 min.
- Using the robotic device, the mean radiation dose rate to the hands of the operating physician was significantly reduced from 0.95 mSv/h to 0.14 mSv/h (-85%).
- Using the robotic device, the mean radiation dose rate to the head of the operating physician was significantly reduced from 0.16 mSv/h to 0.08 mSv/h (-50%).
- Using the robotic device, the mean radiation dose rate to the dominant arm of the operating physician was significantly reduced from 0.12 mSv/h to 0.05 mSv/h (-42%).

## Background

Fluoroscopy-guided puncture is commonly performed for precisely targeting and placing needles and guidewires during different interventional procedures. The use of fluoroscopy results in non-negligible radiation exposure to the operating physician [1–4]. The effective dose range varied from microSievert to mSv per procedure. Radiation doses to the patients, operators, and the assistants had been investigated in different studies [5–9]; other studies specifically investigated the radiation dose to the hands of the operating physician [10–14].

This is a relevant occupational issue when we take in consideration that specialised physicians, in particular interventional radiologists, perform fluoroscopy-guided interventions on a daily basis. The dose limit for various organs was defined by the International Commission on Radiological Protection [15]. In the case of occupational exposure, the average value of effective dose limit is 20 mSv/year, with an absolute limit of 100 mSv over 5 years.

Radiation dose received by the operating physician during fluoroscopy depends upon various factors such as fluoroscopy time, protective shielding against radiation, closeness to the x-ray source, position of the x-ray source, and awareness about radiation exposure. In particular, large variation in the effective dose received by the operating physician when performing arterial puncturing is strongly depending on closeness to the x-ray source.

Passive robots referred to the type of robots that do not use actuators for the motion, with all the joints moved manually. In the medical field, passive robots helped the physician to manually operate a robot by himself rather than operating the motors by the help of joysticks as in the case of active robots. Passive robotics could be a valuable tool for fluoroscopy-guided arterial puncture because it enabled a stable needle control by the operating physician at greater distance from the x-ray source.

Thus, the main aim of this study was to compare radiation doses to different body regions, while performing arterial puncture with and without a passive robotic device.

## Methods

This study was approved by the Animal Ethical Committee of Seoul National University Bundang Hospital (code of approval: BA1708-230/075-01) and was performed in accordance with the Guide for the Care and Use of Laboratory Animals from the Institute of Laboratory Animals Resources [16].

### Design of the passive robot

**Fig. 1:**
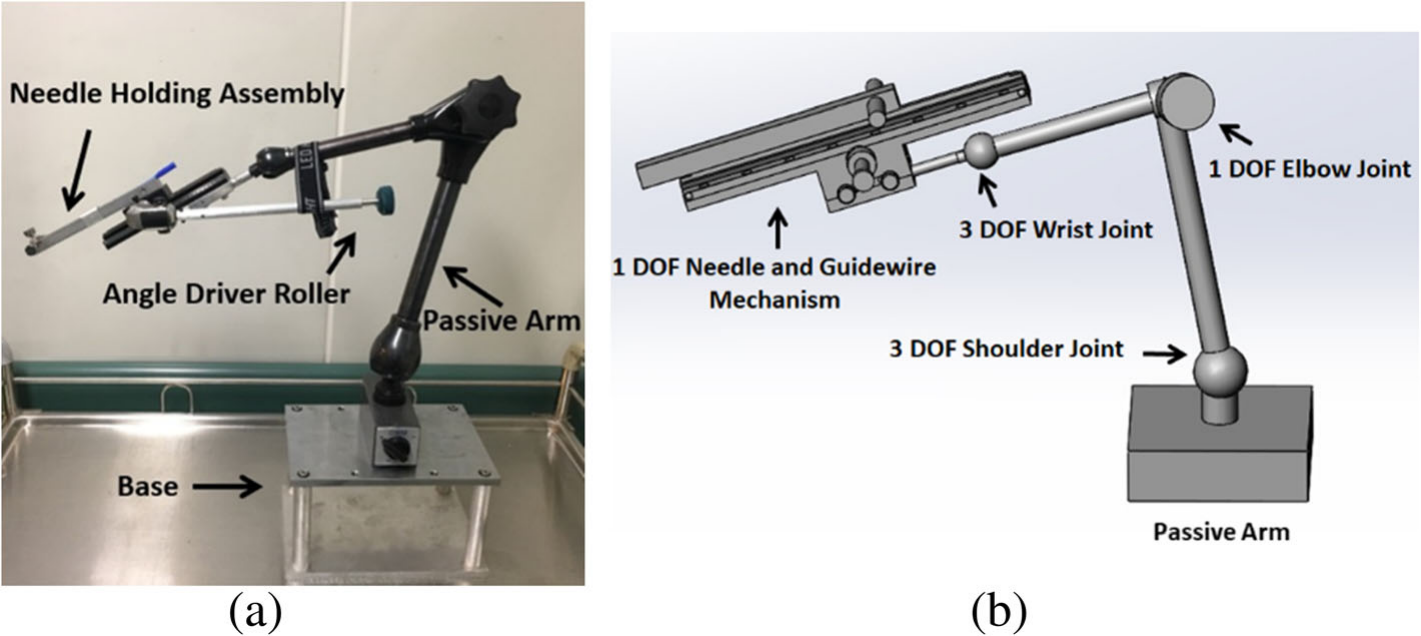
Passive arm and needle holding assembly. **a** Seven-degree of freedom (DoF) passive arm with 1-DoF needle holding assembly. Long angle driver roller allows the physician to perform arterial puncturing away from the x-ray source. Base could be easily fixed on the patient bed. **b** Three-dimensional model of the passive arm and needle holding assembly

The robotic mechanism consisted of a 7-degree of freedom (DoF) passive arm and 1-DoF needle holding assembly as shown in Fig. 1a. The three-dimensional model of the robotic mechanism is shown in Fig. 1b. We employed a 7-DoF commercially available passive arm (MA60003, NOGA Engineering & Technology, Nazareth, Israel). All 7 DoF of the passive arm could be locked at once by locking the knob.

To operate the device without exposing the physician’s hand, a needle holding assembly was designed. The needle holding assembly consisted of a platform, a linear stage for needle insertion, a needle holding aluminium rod, and a roller driving the needle. This device enabled the physician to move the needle easily without exposing herself/himself to x-rays. After positioning the needle to the target blood vessel, the roller could be operated at different configurations to give the physician ease in manoeuvring the needle tip.

**Fig. 2:**
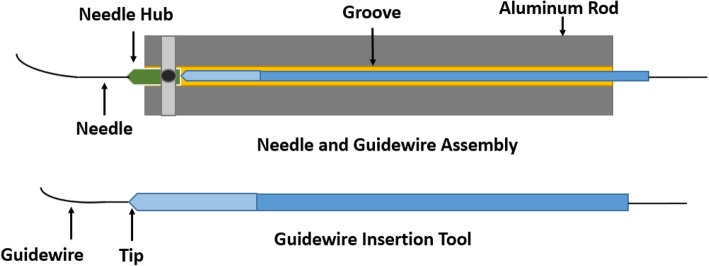
Needle and guidewire holder. The guidewire was inserted inside the guidewire insertion tool. The guidewire insertion tool was placed in the groove of the needle holding assembly. The tip of the guidewire insertion tool was inserted inside the hub of the needle

The linear stage provided forward and backward motion. Needle holding aluminium rod was attached to the linear stage. The hub of the needle was attached to the needle holder assembly by bolts in such a way that the needle remained fixed to the holder during the interaction of the needle with the skin. The needle holder was designed in such a way so that different types of needles can be attached to the holder. Two sideways bolts for needle attachment also helped the physician in accurately aligning the needle to the targeted blood vessel during fluoroscopy-guided arterial puncturing. The third bolt tightened the hub of the needle firmly. For the insertion of a guidewire tool in the hub of the needle, a small groove had been carved out inside the needle holder assembly (Fig. 2). The guidewire was inserted inside the tool. The guidewire insertion tool was designed in such a manner that it would not allow the flexible end of the guidewire to bend. Buckling of the flexible tip of the guidewire could be avoided if the tip of the guidewire insertion tool was inserted properly into the hub of the needle. When the needle was inserted to the targeted blood vessel, blood came out from the hub, indicating the proper insertion of the needle tip. After the insertion of the guidewire, the needle could be removed easily by retracting the 7-DoF passive articulated arm. The base of the device was designed in such a way that it could be easily attached to the patient’s bed with the help of a clamp. It could also be attached to the table next to the bed according to the ease of the physician.

**Fig. 3:**
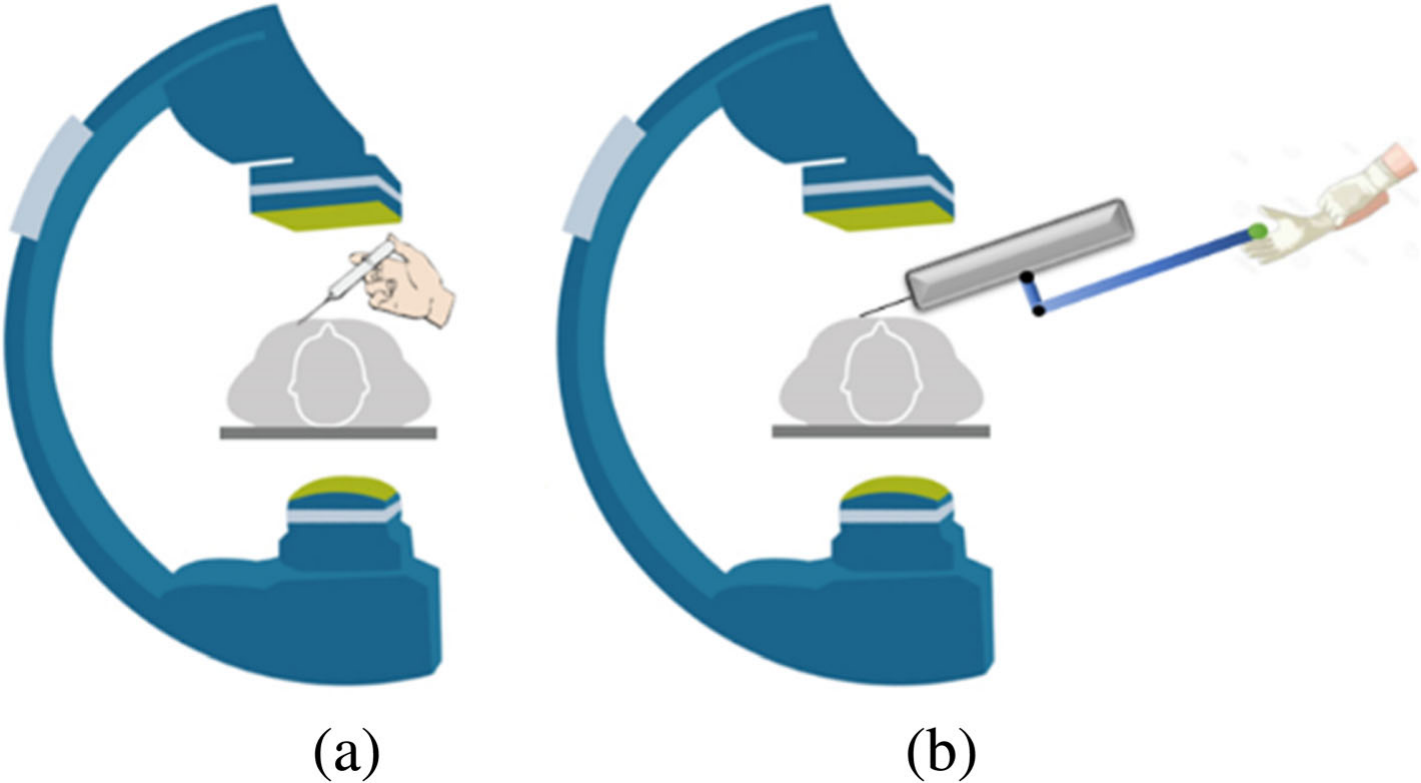
Position of the hand with and without the device. On the left (a), without the device, the physician’s hand is directly exposed to radiation. On the right (b), with the device, the physician’s hand can stay away from the x-ray source

Figure 3a shows the position of the operator’s hand without the device while Fig. 3b shows the position of the operator’s hand with the device. The use of the device allowed the operator to keep his hand away from the x-ray source.

### Animal preparation

The day before the experiment, male crossbred swine (*n* = 15; weight, from 17 to 35 kg) were fed with aspirin (300 mg) and clopidogrel (300 mg). On the experiment day, the swine were premedicated with atropine sulphate (0.05 mg/kg, intramuscularly) and subsequently anaesthetised with Zoletil (5 mg/kg) and Xylazine (4.4 mg/kg) intramuscularly, intubated, and ventilated with room air and isoflurane. We inserted a 6-Fr sheath via the right carotid artery by ultrasound-guided puncture. The animals received heparin (5000 U) intravenously prior to femoral artery digital subtraction angiography.

### Fluoroscopy-guided femoral arterial puncture

A total of 30 fluoroscopy-guided punctures were performed, 15 using the robotic device and 15 without the device by an interventional cardiologist with a 10-year experience, targeting both femoral arteries in the 15 pigs. The mobile fluoroscopy system was Philips BV Pulsera (Philips Medical Systems, Bothell, USA). We selected both femoral arteries with a diagnostic catheter (Judkins right 4, Gifu, Japan) via the right carotid artery and performed angiography to guide the femoral puncture using the Seldinger technique.

Details on the use of the passive robotic device have been described in the previous section describing the design of the passive robot.

### Measurement of radiation exposure

The radiation dose was measured using dosimeters that were attached to the different unprotected parts of the operating physician (Ray 3000, Kedian, Jining, China), and was used to measure the radiation. The dosimeters were attached to the dominant hand, dominant arm, and head of both the operating physician and the assistant animal technician (nurse). In this study, all the dose rates were measured in mSv per hour.

Two parameters were selected to analyse the effectiveness of the device: the success rate of the insertion procedure and the complication level, the latter including three sub-categories which were haematoma (collection of blood outside the vessel); dissection (tear of the vessel wall, which allows blood to separate the wall layers); and occlusion (a blockage of the vessel, usually by a clot or a severe dissection).

### Statistical analysis

Continuous variables were described as mean ± standard deviation (SD), taking into consideration their normal or near normal distribution, confirmed by Shapiro-Wilk test (*p =* 0.9003). As a consequence, the comparison between the means of the two groups was evaluated by Student’s *t* test. A two-sided probability value of < 0.05 was considered indicative of a statistically significant difference. The success rates were presented as percentages with their 95% confidence intervals, calculated according to the binomial distribution. Statistical analysis was performed using R program version 3.1.0.

## Results

Table 1 shows mean radiation dose rates for the arterial puncturing procedures performed by the operating physicians with and without the device. The maximum radiation exposure was measured on the dominant hand without using the device because the hands were directly exposed to radiation. The mean radiation dose rates for the dominant hand of the operating physician during arterial puncturing manually without any device were 0.95 mSv/h. Other unprotected parts like the head and arms were also exposed, and their dose rate was significantly (*p* <  0.001) lower as compared to hands.

**Table 1:** Radiation dose rate (mSv/hour) for the operating physician with and without the passive robotic device

	Dominant hand (*n* = 15)	Head (*n* = 15)	Dominant arm (*n* = 15)
Dose rate without the device (mean ± SD)	0.95 ± 0.25	0.16 ± 0.02	0.12 ± 0.02
Dose rate with the device (mean ± SD)	0.14 ± 0.06	0.08 ± 0.01	0.07 ± 0.01
*p* value	< 0.001	< 0.001	< 0.001
Relative decrease	- 85.2	- 50.0	- 41.6

*SD* standard deviation

The mean radiation dose rates for the dominant hand of the physician during arterial puncturing with the device were 0.14 mSv/h. With the device, an 85% reduction in the operating physician’s hand exposure was observed. In the case of the head and arm, mean dose rates were reduced from 0.16 mSv/h to 0.08 mSv/h and 0.12 mSv/h to 0.07 mSv/h, respectively (see Table 1).

Table 2 shows the results concerning efficacy and safety measurements. The success rate of performing arterial puncturing was 15/15 (100%, 95% CI 0.78–1.00) both using or not using the robotic device. The mean exposure time with the device had been reduced to 4.3 min while without the device, it was 4.5 min. In this study, two dissections and two haematomas occurred without the device while two dissections occurred when the physician performed arterial puncturing using the device.

**Table 2:** Fluoroscopy time, success rate, and complications with and without the passive robotic device

	Without device (*n* = 15)	With device (*n* = 15)
Fluoroscopy time (min, mean ± SD)	4.5 ± 0.15*	4.3 ± 0.11*
Success rate	15/15 (100%, 95% CI 0.78–1.00)	15/15 (100%, 95% CI 0.78–1.00)
Complications	2 haematomas 2 dissections	2 dissections

*SD* standard deviation, *CI* confidence interval

*p = 0.002

**Fig. 4:**
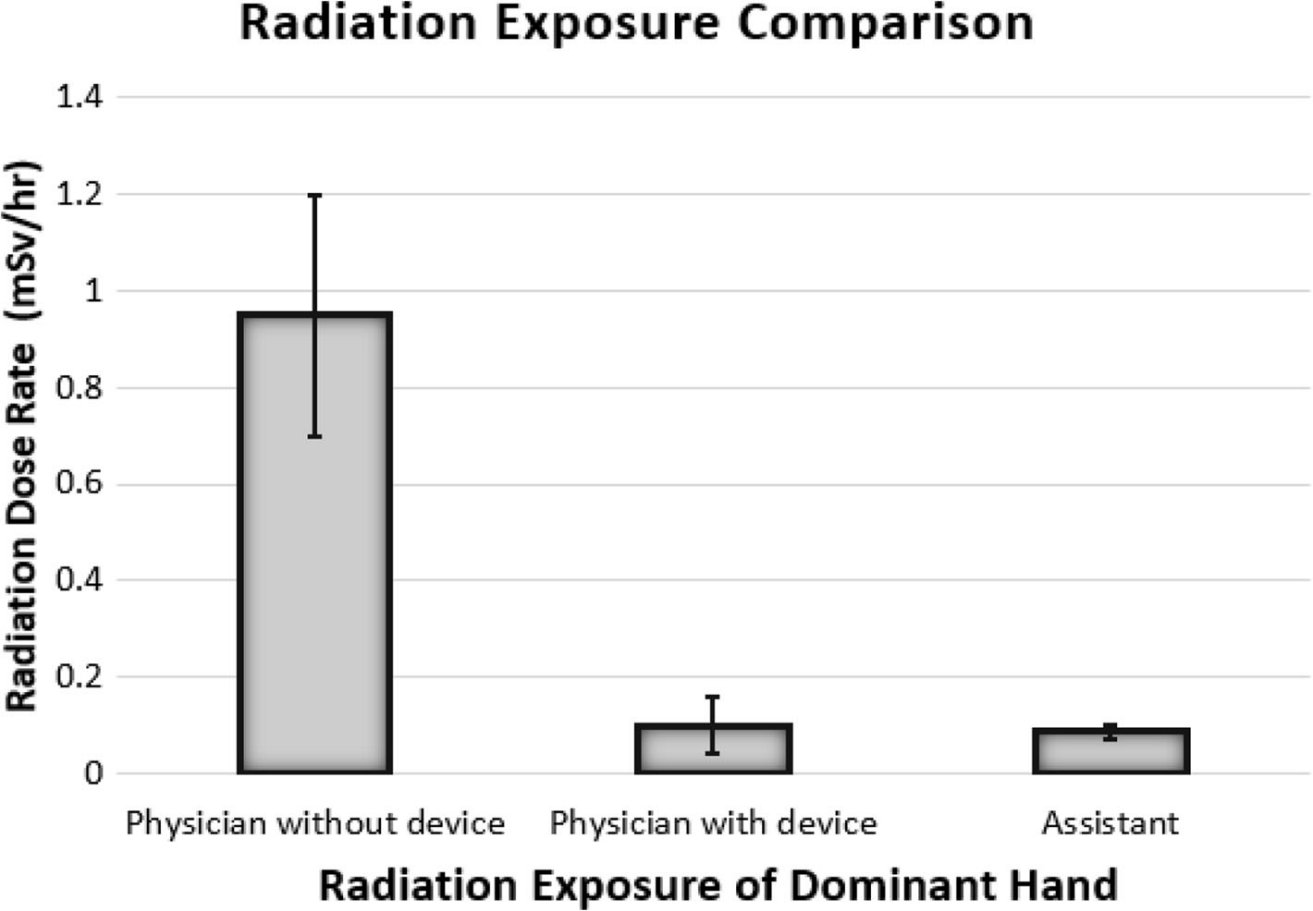
Mean radiation exposure of the dominant hand (mSv/h) with standard deviation. Without the device, the operating physician was exposed to a higher radiation dose rate than that observed when using the device (*p* < 0.001), the latter being almost similar to the radiation exposure of the assistant (*p* = 0.042)

Table 3 shows radiation dose rates for the assistant helping the operating physician: the mean radiation exposure was significantly lower than that of the operating physician for both cases when the physician performs puncturing procedures with or without the device (*p* = 0.003 and *p* < 0.001, respectively). Figure 4 shows the mean radiation exposure rate (mSv per hour) of the dominant hand. With the help of the device, the physician would get almost the same amount of radiation dose rate as that of the assistant.

**Table 3:** Radiation dose rate (mSv/hour) for the assistant with and without the passive robotic device

Location of dosimeter	Dose rate with the device (mean ± SD, *n* = 15)	Dose rate without the device (mean ± SD, *n* = 15)	*p* value
Dominant hand	0.08 ± 0.02	0.09 ± 0.01	0.042
Head	0.08 ± 0.01	0.09 ± 0.01	0.021
Dominant arm	0.03 ± 0.01	0.04 ± 0.01	0.019

*SD* standard deviation

## Discussion

The goal of the study was to compare the x-ray dose rates for the operating physician and the assistant when performing fluoroscopy-guided arterial puncturing with or without a passive robotic device. It was observed that the operating physician was significantly more exposed to radiation as compared to the assistant. Amongst the body parts of the operating physician, the hands received significantly more radiation as compared to the head and arms. With the use of passive robotic device, an 85% decrease in radiation exposure rate of the hands was observed for the operating physician. The success rate of performing the arterial puncturing with the device was 100% (*n* = 15). The mean fluoroscopy time was significantly (*p* = 0.002) reduced from 4 min and 30 s without the device to 4 min and 18 s with the device.

Two dissections when using the device while two dissections and two haematomas occurred when not using the device. Of note, the occurrence of complications as a consequence of arterial puncturing in humans has been reported to be very low, *i.e.* less than 0.5% [17]. However, dissections and haematomas occur more frequently in animal models due to smaller arterial size and higher susceptibility of the arterial wall to complications.

We observed that the skill of operating the passive robotic device played an important role in the reduction of mean fluoroscopic exposure time. Initially, the operating physician took longer time to perform arterial puncturing using the device, but as he had developed the skill of operating the device, the mean fluoroscopic exposure time had been reduced and it took the physician three experiments to become familiar with the use of the device.

When the operating physician performed the arterial puncturing using the robotic device, the hand of the physician remained far from the x-ray source. As a result, the radiation exposure to hands was significantly reduced.

Physicians utilise different ways to protect themselves from ionising radiation either by wearing thyroid collar and lead apron or by optimising the use of fluoroscopic devices and, in some cases, proper positioning of x-ray source and operating fluoroscopic devices in pulsed mode [18–20]. However, the physicians and assistants are still exposed to ionising radiation even after using different protective techniques. Robot-assisted vertebral body augmentation had reduced the radiation exposure for both the surgeon and medical staff [21]. A recent position paper on medical radiation of the European Society of Cardiology [22] urged technological innovations in the field to enhance the safety of the doctors working in the cardiac catheterisation laboratory. The robotic mechanism would be a way to the innovation.

To solve this problem, a passive robotic mechanism had been designed that helped the physician to stand and perform arterial puncturing away from the close vicinity of the x-ray source. As a result, significant reduction of radiation happened for not only the hands of the operating physician but also for his other unprotected body parts.

One limitation of this study is the limited sample size. However, the reduction in radiation exposure associated with the use of the robotic device resulted to be significant. In addition, we should consider the passive limitation of the robotic mechanism: the physician had to operate it using his hands and could not operate remotely as in the case of active robotic mechanisms.

In conclusion, in this study on a swine model, the use of a passive robotic mechanism for arterial puncturing and guidewire insertion had significantly reduced radiation exposure for not only the hands but also for the overall body of the operating physician.

## Abbreviations

DoF: Degree of freedom
SD: Standard deviation
